# Selection and screening of drought tolerant high yielding chickpea genotypes based on physio-biochemical indices and multi-environmental yield trials

**DOI:** 10.1186/s12870-020-02381-9

**Published:** 2020-04-17

**Authors:** Tariq Mahmud Shah, Muhammad Imran, Babar Manzoor Atta, M. Yasin Ashraf, Amjad Hameed, Irem Waqar, M. Shafiq, Khalid Hussain, M. Naveed, M. Aslam, Muhammad Amir Maqbool

**Affiliations:** 1 Plant Breeding & Genetics Division, Nuclear Institute for Agriculture and Biology (NIAB), Jhang Road, Faisalabad, Pakistan; 2 Nuclear Institute for Food and Agriculture (NIFA), Peshawar, Pakistan; 3 Institute of Biology and Biotechnology (IMBB), the University of Lahore, Lahore, Pakistan; 4 Pulses Research Institute, AARI Faisalabad, Faisalabad, Pakistan; 5 Arid Zone Research Institute (AZRI), Bhakkar, Pakistan; 6 University of Agriculture, Faisalabad (U.A.F.), Faisalabad, Pakistan

**Keywords:** Chickpea, Drought tolerance, Physiological traits, And screening criteria

## Abstract

**Background:**

Chickpea is one of the major legume crops being cultivated in the arid and semi-arid regions of Pakistan. It is mainly grown on the marginal areas where, terminal drought stress is one of the serious threats to its productivity. For defining the appropriate selection criteria for screening drought tolerant chickpea genotypes, present study was conducted. Distinct chickpea germplasm was collected from different pulses breeding institutes of Pakistan and evaluated for drought tolerance at germination and early seedling stages, furthermore, at late vegetative growth stages physiochemical traits and multi-environment yield performance were also tested.

**Results:**

Chickpea genotypes under different environments, were significantly varied for different seedling traits, physio-chemical attributes and seed yield. Genotypes showing drought tolerance by performing better at an early seedling stages were not correspondingly high yielding. Screening for drought tolerance on seed yield basis is the most appropriate trait to develop the drought tolerant as well as high yielding chickpea genotypes. Results confirmed that traits of early growth stages were not reflecting the drought tolerance at terminal growth stages and also did not confer high yielding. NIAB-rain fed environment proved ideal in nature to screen the chickpea genotypes whereas, NIAB-lysimeter and Kalur Kot was least effective for selecting genotypes with high seed yield. Genotypes D0091–10, K010–10, D0085–10, K005–10, D0078–10, 08AG016, 08AG004, D0080–10, 09AG002, K002–10 and D0099–10 were high yielding and drought tolerant based on their performance across multiple hotspot environments.

**Conclusions:**

The selected genotypes are intended for further evaluation for varietal approval to recommend for general cultivation on farmer fields in drought hit areas of Pakistan. Among physio-biochemical traits, higher proline, glycine betain, RWC and CMS were reflecting the higher capability to tolerate the drought stress in chickpea. Drought sensitive genotypes (K0037–10, 2204, K0052–10, 09AG015, K0042–10, CM709/06, K0068–10, K004–10, K0026–10 and K0063–10) were also identified in present study which were resourceful asset for using as contrasting parents in hybridization programs. To our knowledge, this is first report using an integrated approach involving, physio-biochemical indices, and multi-environmental yield trials, for comparison, screening and selection of chickpea genotypes for drought tolerance.

## Background

Crop plants are subjugated by wide spectrum of biotic and abiotic stresses which seriously hinders their optimal growth, development and reproduction. Scientific studies have proven that water deficiency adversely affected the crop growth and productivity that is a serious threat for agriculture [34]. Presently about one-third of world’s population is living in water-deficit regions whereas and it is predicted that severity, duration and frequentness of drought stress is expected to increase due to elevated CO_2_ in atmosphere and climatic changes [3]. Resilience of the legume crops in current prevailing weather extremities could be the futuristic adaptation in more severe climatic conditions [13].

Chickpea (*Cicer arietinum* L.) the second most important grain legume cultivated by resource poor farmers in the arid and semi-arid regions of the world especially Pakistan [27, 42]. It is mostly grown under rainfed conditions. Availability of water in rainfed regions is either in form of stored soil moisture in subtropical environment with summer-prevalent rainfall or only at the time of seasonal rainfall. In these conditions rainfed chickpea plantations encounters the serious yield losses due to terminal drought stress [40, 43, 44]. Drought stress is one of the major constraints for chickpea, which causes up to 50% yield losses [42]. Drought is a complex abiotic stress, which affects various physiological and biochemical processes of crop plants. These disturbances cause growth reduction, decrease in chlorophyll contents, decrease in ascorbic acid, increase in proline accumulation, hydrogen peroxide [14, 27, 28]. These attributes can be used to screen genotypes for drought tolerance. Moreover, high relative water content (RWC) and low excised leaf water loss (ELWL) was related to drought resistance [4, 15]. So, all these are being used as screening tools in different crops.

Understanding of genetic manipulation to encounter the drought stress like, drought escape, drought avoidance and drought tolerance is necessary to develop drought tolerant or resistant crop plant [27, 39, 41]. Drought tolerance is complex phenomenon established through biosynthesis of osmolytes or compatible solutes, osmoprotectants and secondary metabolites and adjusting water relations [22, 27, 29]. Sustainable development of drought tolerant genotypes is necessary to fulfill the food demands of ever growing world population which possible through continuous evaluation of genotypes for stress tolerance in drought prone regions and selection of genotypes on the basis of yield performance [20, 30]. Key objectives of this study were to explore the drought tolerant chickpea genotypes with high yield performance for releasing as commercial cultivar.

## Methods

### Experimental material and Germplasm collection

Present study was comprised of the 55 chickpea genotypes (Table 1), collected from three research institutes including Nuclear Institute for Agriculture and Biology (NIAB), Faisalabad, Punjab, Pakistan, Arid Zone Research Institute (AZRI), Bakhar, Punjab, Pakistan and Pulses Research Institute (PRI), AARI, Faisalabad, Punjab, Pakistan. Performance of these chickpea genotypes for drought tolerance was evaluated through different studies to select high yielding genotypes for commercial cultivation. Experiments were conducted in growth chamber to determine physiological & biochemical index and in fields to located in hot spot regions of the country.

**Table 1:** List of chickpea mutant genotypes collected from different research institutes

Sr. No.	Mutant No.	Origin	Sr. No.	Mutant No.	Origin	Sr. No.	Mutant No.	Origin
1	CM709/06	NIAB	20	K0013–10	PRI	39	09AG019	AZRI
2	CM776/06	NIAB	21	K0014–10	PRI	40	09AG015	AZRI
3	CM1004/06	NIAB	22	K0016–10	PRI	41	08AG004	AZRI
4	CM687/06	NIAB	23	K0017–10	PRI	42	08AG016	AZRI
5	CM731/06	NIAB	24	K0019–10	PRI	43	09AG002	AZRI
6	CM795/06	NIAB	25	K0024–10	PRI	44	09AG006	AZRI
7	CM848/06	NIAB	26	K0026–10	PRI	45	08AG015	AZRI
8	CM1528/03	NIAB	27	K0027–10	PRI	46	D0078–10	PRI
9	2124	PGRI	28	K0030–10	PRI	47	D0079–10	PRI
10	09AK055	AZRI	29	K0031–10	PRI	48	D0080–10	PRI
11	2175	PGRI	30	K0032–10	PRI	49	D0081–10	PRI
12	2204	PGRI	31	K0037–10	PRI	50	D0085–10	PRI
13	K002–10	PRI	32	K0042–10	PRI	51	D0091–10	PRI
14	K003–10	PRI	33	K0049–10	PRI	52	D0094–10	PRI
15	K004–10	PRI	34	K0052–10	PRI	53	D0096–10	PRI
16	K005–10	PRI	35	K0063–10	PRI	54	D0097–10	PRI
17	K008–10	PRI	36	K0066–10	PRI	55	D0099–10	PRI
18	K010–10	PRI	37	K0068–10	PRI			
19	K012–10	PRI	38	K0069–10	PRI			

*NIAB* Nuclear Institute for Agriculture and Biology, *AZRI* Faisalabad; Arid Zone Research Institute, *PRI* Bakhar; Pulses Research Institute, *AARI* Faisalabad

### Growth chamber evaluation

For physiological indices studies were conducted in growth room under controlled conditions. Ten seeds of each genotype were sown in glass bowls containing polyethylene glycol (PEG-6000) of-0.60MPa solution prepared by dissolving 180 g of PEG in 1 l of distilled water [5, 31]. Seeds were placed on gauze in glass bowls (dia. 14 cm x depth 8 cm) containing 70 ml of the test solution. A control of these experiments was also maintained with 0.00 MPa. These bowls were kept in growth chamber at running 30/25 °C (day/night) temperature. Darkness was maintained during the experiment upto the seed germination. Germination data was recorded when the radicles were of 5mmin length. The germination stress tolerance index was calculated by determining the promptness index (P.I.) following the George [18].

P.l. = nd_2_ (1.00) + nd_4_ (0.75) + nd_6_ (0.50) + nd_8_ (0.25).

Where, nd_2_, nd_4_, nd_6_ and nd_8_were seeds germinated on the 2nd, 4th, 6^th^and 8th day of sowing, respectively.

Germination stress tolerance index (GSI) was calculated as described by [5];

$$ \mathrm{GSI}=\frac{P.I.\kern0.5em of\ stress\ seeds}{P.I.\kern0.5em of\ control\ seeds}\times 100 $$

The seedlings were allowed grow for14 days, after harvesting the data for shoot and root lengths were recorded. After recording fresh weights of shoot and root, they were dried at 70 °C for 48 h in an oven and their dry weights were estimated on electrical digital balance. This data were used to calculate root and shoot length stress tolerance indices (RLSI and SLSI, respectively), fresh and dry weight stress tolerance indices (FWSI and DWSI, respectively) as under;

$$ \mathrm{RLSI}=\frac{\mathrm{Root}\ \mathrm{length}\ \mathrm{of}\ \mathrm{stressed}\ \left(-0.6\ \mathrm{MPa}\right)\ \mathrm{plants}\ }{\mathrm{Root}\ \mathrm{length}\ \mathrm{of}\ \mathrm{control}\ \left(0.0\ \mathrm{MPa}\right)\ \mathrm{plants}\kern0.75em }\times 100 $$

$$ \mathrm{SLSI}=\frac{\mathrm{Shoot}\ \mathrm{length}\ \mathrm{of}\ \mathrm{stressed}\ \left(-0.6\ \mathrm{MPa}\right)\ \mathrm{plants}\kern0.75em }{\mathrm{Shoot}\ \mathrm{length}\ \mathrm{of}\ \mathrm{control}\ \left(0.0\ \mathrm{MPa}\right)\ \mathrm{plants}}\times 100 $$

$$ \mathrm{FWSI}=\frac{\mathrm{Fresh}\ \mathrm{weight}\ \mathrm{of}\ \mathrm{stressed}\ \left(-0.6\ \mathrm{MPa}\right)\ \mathrm{plants}}{\mathrm{Fresh}\ \mathrm{weight}\ \mathrm{of}\ \mathrm{control}\ \left(0.0\ \mathrm{MPa}\right)\ \mathrm{plants}}\times 100 $$

$$ \mathrm{DWSI}=\frac{\mathrm{Dry}\ \mathrm{weight}\ \mathrm{of}\ \mathrm{stressed}\ \left(-0.6\ \mathrm{MPa}\right)\ \mathrm{plants}}{\mathrm{Dry}\ \mathrm{weight}\ \mathrm{of}\ \mathrm{control}\ \left(0.0\ \mathrm{MPa}\right)\ \mathrm{plants}}\times 100 $$

### Cell membrane stability

Cell membrane stability (CMS) was determined according to the procedure developed by [37]. Seeds of different genotypes were sown in two cemented tanks (3 × 3 m) filled with medium textured soil. After the period of 4 weeks water was removed from one tank while normal irrigation was applied to the other tank up to 4 weeks. Leaf samples were collected from plants grown in these two conditions. Leaf discs (1 g) of 0.5 cm size were cut from the fully developed upper leaflet placed in a 50 ml test tube which were washed three times with deionized water before imbedding in test solution of PEG. These leaflet discs were inundated in 30 ml of 40% PEG-6000 solution (T_1_) and in deionized water as a control (C_1_) then both were incubated at 10 °C for 24 h. After which the leaf discs were again washed three times with deionized water and floated in 30 ml deionized water for at 10 °C for 24 h. Then electrical conductivity (EC) of the liquid was then measured with conductivity meter. After which leaf discs l in the same solution were autoclaving for 20 min and EC was again measured at 25 °C (T_2_, C_2_) Cell membrane stability was calculated as under;

$$ \mathrm{Percent}\ \mathrm{Injury}=\left\{1-\frac{\left(1-\frac{T1}{T2}\right)}{\left(1-\frac{C1}{C2}\right)}\right\}\times 100 $$

CMS = 100- Percent Injury.

### Evaluation of physiological and biochemical markers

From the field studies at NIAB, Faisalabad, Pakistan, leaf samples were collected to determine different physiological and biochemical attributes necessary to estimate the genotypic variability. Following biochemical and physiological parameters were estimated:

### Excised leaf water loss

After getting weight of leaves, excised leaf water loss (ELWL) was recorded in three steps,

1. Fresh weight (immediately after sampling)
2. After drying in an incubator at 28 °C at 50% R.H.(Relative humidity) for 6 h
3. Re-drying in an oven for 24 h at 70 °C as suggested by Clarke and Townley-Smith [12].

Excised leaf water loss was calculated from the following formula;

$$ \mathrm{Excised}\ \mathrm{leaf}\ \mathrm{water}\ \mathrm{loss}=\frac{Fresh\ Weight- Weight\ after\ 6\  hrs}{Fresh\ Weight-\kern0.5em Dry\  Weight} \times 100 $$

### Relative water contents

Relative water contents (RWC) is possibly the proper measure of plant water status in terms of the physiological outcome of cellular water deficit. Methods of Barrs and Weatherley [9] were used to determine the RWC. For this parameter 100 mg leaf material was taken and kept in double distilled water in a petridish for 2 h to make the leaf tissue turgid. The turgid weight and dry weight of the leaf materials was measured and RWC was calculated with the help of formula given below.

$$ \mathrm{RWC}\ \left(\%\right)=\frac{\left( Fresh\ Weight\ of\ Sample- Dry\  Weight\ of\ Sample\right)}{Turgid\ Weight\ of\ Sample-\kern0.5em Dry\  Weight\ of\ Sample}\times 100 $$

### Proline content

Fresh chickpea leaves were used to determine proline contents according to the method of Bates et al. [10]. The fresh leaf material (1 g) was homogenized in 3% aqueous solution of sulphosalicylic acid (10 ml). Leaf samples were centrifuged at speed of 12,000 rpm in centrifuge machine for 10 min and removed the residue. The supernatant was used (1 ml) was allowed to react with 1 ml of acid-ninhydrin (1.25 g ninhydrine in 30 ml glacial acetic acid and 20 ml 6 *M* orthophosphoric acid) p and 1 ml of glacialacetic acid in a test tube (25 ml) for 1 h at 100 °C. Reaction mixture was extracted with 10 mL toluene, which was mixed vigorously by passing a continuous stream of air for 1–2 min. Toluene was aspirated from chromophore. Aqueous phase was taken, warmed at room temperature and read at 520 nm absorbance using toluene as a blank. The proline concentration was estimated from a standard curve developed with different concentration of D-proline using following formula.

**(**μmole **proline g**^**− 1**^**fresh weight =**

**(**μg **proline mL**^**− 1**^**x mL of toluene/115.5)/(g of sample/10)**

### Nitrate reductase activity

Nitrate reductase activity (NRA) in leaves of chickpea was determined according to reported method [32] in which potassium nitrate act as substrate. Enzyme from leaves was extracted with0.02 M phosphate buffer (pH 7.0). Fresh plant material (0.5 g) was homogenized in4.5 ml phosphate buffer (pH 7.0) and 0.5 ml of 0.02 M KNO_3_. Reaction mixture was incubated at 32 °C in dark for 1 h. The reaction was terminated by adding 1 ml of 1% sulfanilamide prepared in 2 N HCl and then 1 ml of 0.02% of aqueous solution of N 1-Naphthyl-ethylene diaminedihydrochloride was added. The optical density of the reaction mixture was measured at 542 nm on a spectrophotometer. Activity of NRA was determined as μmoles of NO_2_ g^− 1^F.W h^− 1^ by using standard curve developed with NaNO_2_.

### Glycine betaine contents

Glycine betaine in leaf samples of chickpea genotypes were determined by using the protocol recommended by [19]. Leaf samples of 0.5 g were finely ground and then vortexed with 20 ml deionized water for 48 h at 25oC. Filtrate from this solution was mixed with equal volume of 2 N sulphuric acid (1:1 ratio). Aliquot of 0.5 ml volume was taken and cooled for 1 h in ice water. Mixture was vortexed after the addition of 0.2 ml cold potassium iodide-iodine reagents. These samples were stored at 0-4 °C for 16 h and subsequently these were centrifuged 10,000 rpm for 15 min at 0 °C. Supernatant was discarded and then preiodite crystals were dissolved in 9 ml of 1, 2-dichloro ethane. Samples were incubated for the period of 2.0 to 2.5 h and then absorbance was determined at 365 nm with UV-visible spectrophotometer. Standard solutions of glycine betaine (50–200 μg/ml) were prepared in 2 N sulphuric acid and then subjected to spectrophotometer absorbance similar to the procedure followed for samples.

### Multi-environment evaluation for seed yield

These 55 chickpea genotypes were also evaluated for yield performance across different environments for evaluating the genotype × environment interaction for grain yield. The field experiment was conducted in randomized complete block design with three replications under each environment. These targeted environments were NIAB-lysimeter, NIAB-irrigated, NIAB-rainfed, *AZRI*, AARI and Kaloorkot. NIAB-lysimeter, NIAB-irrigated and NIAB-rainfed environments were developed at Nuclear Institute for Agriculture and Biology, Faisalabad, by managing irrigation pattern. NIAB-lysimeter environment (triplicate) was given irrigation only at the soil preparation prior to the sowing and access of the rain water was also prevented by developing protective shelters [35]. NIAB-irrigated environment was generated by provision of only two irrigations i.e. during field bed preparation and initiation of flowering. Rainfall was also accessible to the fields for NIAB-irrigated environment. NIAB-rainfed environment was demonstrated as only irrigation was provided at field bed preparation and second water source was only rain. Whereas, other three environments belongs to different hotspot locations for chickpea cultivation and uncertainty of water availability. Water source at these three locations was only rainfall except the initial irrigation for soil bed preparations. For comparison of yield performance well known commercial cultivars i.e. CM-2008, Noor-2009, Pb 2008 and Thall 2011 were used as local checks. Plot size was1.5 m × 1.2 m, while P × P and R × R distance was 30 cm and 15 cm, respectively. All other standard agronomic practices were followed across all of the subjected environments from sowing to harvesting except for provision of irrigation water. Upon harvesting yield per plot was calculated as g plot^− 1^ and converted to kg ha^−1^for subsequent analysis.

### Statistical analysis

Data acquired from growth chamber investigation was subjected to analysis of variance (ANOVA) under completely randomized design (CRD) with three replications [36]. Data acquired for biochemical and physiological parameters from field experiment was subjected to analysis of variance under randomized complete block design (RCBD). All of the targeted six different environments were separately subjected to analysis of variance to determine the significance of environmental effects on genotypic differences. Multi-environment yield data for different chickpea genotypes was subjected to analysis of variance under factorial treatment structure where, genotypes and environments were two different factors. Genotypic selection was most authenticated by use of Principal Component Analysis (PCA) based biplots ([22] a, b [7];) for these studies separately and combined. GGE biplot analysis was used for assessment of pattern for genotype × environment interaction. Depiction of average environment, best environment, average performing genotype and best genotypes is facilitated by GGE biplot analysis across diverse environments [45, 46].

## Results

### Partitioning of variability and assessment of significance for differences

Variability for growth chamber evaluation was partitioned into various components to determine the significance of genotypic differences for growth indices. Highly significant genotypic differences were observed for GSI, DWSI, FWSI, RLSI and SLSI in this study (Table 2). Dissection of variability for physiological and biochemical markers showed, the highly significant genotypic differences for CMS, ELWL, Electrolyte leakage, Glycine-betaine, NRA, Proline and RWC (Table 2).ANOVA for each environment separately showed that genotypes were significantly different in their yield performance at AARI, AZRI, K. Kot and NIAB-rainfed. However, genotypes were insignificantly different for yield performance under NIAB-lysimetric and NIAB-irrigated environments (Table 2). Combined ANOVA across the environments showed that genotypes, environments and genotype × environment interaction (GEI) were significantly different for grain yield (Table 3). Significance of GEI showed that performance of the genotypes was different across various environments.

**Table 2:** Analysis of variance for different traits of early growth stages, physio-chemical characteristics and grain yield at different locations Where, RLSI: root length stress tolerance index, SLSI: shoot length stress tolerance index, FWSI: fresh weight stress tolerance index, DWSI: dry weight stress tolerance index, CMS: Cell membrane stability, ELWL: excised leaf water loss, NRA: Nitrite reductase activity, RWC: relative water contents, NIAB: Nuclear Institute for Agriculture and Biology, AARI: Ayub Agricultural Research Institute, K.Kot: Kalur kot farms

**Traits of Early Growth Stages**								
SOV	df	DWSI	FWSI	GSI	RLSI	SLSI		
Mutant	54	1077**	839.42	1077.9**	1405.08**	4448.57**		
Error	110	0.72	1.252	0.77	2.69	2.44		
Total	164							
CV		2.09	3.44	1.03	3.50	2.24		
**Physiological and Biochemical Traits**								
SOV	df	CMS	ELWL	Electrolyte leakage	Glycinebetaine contents	NRA	Proline contents	RWC
Block	2	0.2156	0.3220	0.1898	0.00243	0.2179	0.250	9.940
Mutants	58	59.79**	0.3824**	31.519**	0.3572**	2.2293**	133.774**	589.981**
Error	116	1.2009	0.2243	0.0516	0.00104	0.0827	0.034	15.763
Total	176							
CV		1.29	5.79	4.73	2.03	6.94	2.52	5.64
**Seed Yield under different environments**								
SOV	df	AARI	AZRI	NIAB-Lysimeter	NIAB-Irrigated	NIAB-Rainfed	K. Kot	
Reps	2	150.28	18,708	188.021	28,759	11,254.1	26.730	
Mutants	58	2192.6**	535474**	55.657 ns	7055.1	4836.7*	433.841**	
Error	116	579.63	28,737	90.438	6119.1	3254.4	99.127	
Total	176							
CV		32.52	9.69	101.92	30.30	24.66	21.78

**Table 3:** Analysis of variance for seed yield of chickpea genotypes in multi-environment evaluation

SOV	DF	SS	MS	F	P	Significance
Replications	2	18,206.7	9103.36			
Genotypes (G)	58	6,274,223	108,176	16.57	0.0000	**
Environments (E)	5	3.990E+ 08	7.980E+ 07	12,220.6	0.0000	**
G × E	290	2.563E+ 07	88,374.4	13.53	0.0000	**
Error	706	4,610,027	6529.78			
Total	1061	4.355E+ 08				
CV	20.47					

### Mean comparison

Basic summary statistics and genotypic means were estimated for all studied traits to examine the variability is acquired data. Mean for DWSI was 40.46, that was within the range of 5.85 to 104.32 in growth chamber study. Mean for EWSI was 32.51, that was also within the range of 4.35 to 76.29. Similarly, GSI values were ranged from 33.48 to 133.78 with the mean value of 85.31. RLSI has the mean 46.80 within the range of 8.37 to 113.25. Mean value for SLSI was 69.90 within the range of 17.35 to 257.19. Other statistical parameters like, standard deviation, standard error of mean, First, 2nd and 3rd quartiles for traits studied in growth chamber were also given in Table 4.

**Table 4:** Summary Statistics for different seedling, physio-chemical traits and seed yield

	Mean	Minimum	Maximum	Standard deviation	Standard error of mean	1st quartile	2nd quartile	3rd quartile
**Traits of Early Growth Stages**								
DWSI	40.46	5.85	104.32	18.95	2.51	27.27	38.58	47.68
EWSI	32.51	4.35	76.29	16.73	2.55	20.37	30.26	43.34
GSI	85.31	33.48	133.78	18.96	2.557	75.63	88.70	99.77
RLSI	46.80	8.37	113.25	21.64	2.92	32.56	44.24	53.21
SLSI	69.90	17.35	257.19	38.51	5.19	44.61	59.33	94.12
**Physiological and Biochemical Traits**								
CMS	85.114	75.00	95.47	4.464	0.581	82.83	84.40	95.47
ELWL	8.186	7.67	9.000	0.357	0.046	8.000	8.000	9.00
Electrolyte Leakage	4.799	1.997	20.91	3.241	0.422	2.827	3.452	20.907
Glycinebetaine	1.589	1.00	2.609	0.345	0.0449	1.316	1.614	2.609
NRA	4.095	1.504	5.065	0.927	0.121	4.000	4.403	5.065
Proline	21.36	2.693	71.49	20.43	2.66	6.484	9.292	71.49
RWC	70.46	35.71	120.0	14.03	1.83	64.82	69.09	120.0
**Seed Yield under different environments**								
AARI	1639	679.0	3273.0	602.93	78.49	1192.0	1581.0	1985.0
AZRI	1749	919.0	2793.0	422.48	55.02	1467.0	1652.0	2015.0
K.Kot	1016	400.0	1663.0	284.75	37.07	756.00	1008.0	1215.0
NIAB-lysimeter	206.88	51.00	506.0	96.048	12.51	138.00	193.00	259.00
NIAB-irrigated	2868	1584.0	3898.0	538.60	70.12	2557.0	2833.5	3339.0
NIAB-rainfed	2569.6	1170.0	3439.0	446.26	58.09	2316.0	2511.0	2855.0

Where, *RLSI* Root length stress tolerance index, *SLSI* Shoot length stress tolerance index, *FWSI* Fresh weight stress tolerance index, *DWSI* Dry weight stress tolerance index, *CMS* Cell membrane stability, *ELWL* Excised leaf water loss, *NRA* Nitrite reductase activity, *RWC* Relative water contents, *NIAB* Nuclear Institute for Agriculture and Biology, *AARI* Ayub Agricultural Research Institute, *K.Kot* Kaloor kot farm

Biochemical and physiological traits were also subjected to summary statistics. Mean for CMS was 85.114% with the range of 75.00 to 95.47%. Highest value for ELWL was 9.00% and lowest was 7.67% whereas, mean was 8.19%. Mean for ECCL was 4.799% while minimum value was 1.997% and the highest was 20.91%.Glycinebetaine (GB) has mean value 1.59 μmol/g within the range of 1.00 μmol/g to 2.609 μmol/g dry weight. The lowest value for NRA was 1.504 μmolNO_2_ g^− 1^ fresh weight h^− 1^and the highest value was 5.065 μmolNO_2_ g^− 1^ fresh weight h^− 1^while mean was 4.095μmolNO_2_ g^− 1^ fresh weight h^− 1^. Proline concentrations were ranging from 2.69to 71.49 μmolg^− 1^ fresh weight with the mean value of 21.36μmolg^− 1^ fresh weight. Mean across all genotypes for RWC was 70.46% while the lowest value was 35.71% and the highest was 120.00%. Other statistical parameters like, standard deviation, standard error of mean, 1st, 2nd and 3rd quartiles for traits studied in growth chamber were also given in Table 4.

Means for yield of chickpea genotypes across all of the six different locations were also accessed. Mean yield at AARI was 1639 kg/hac with the range of 679.0 to 3273 kg/hac.0. AZRI produced the mean yield 1749 kg/ha for all genotypes while lowest yield was 919.0 and highest was 2793.0. Mean yield for K. Kot was 1016 with the range of 400.0 to 1663.0. NIAB-lysimter produced the mean yield of 206.88 with the range of 51.00 to 506.0. Mean yield at NIAB-irrigated was 2868 with the range of 1584.0 to 3898.0. NIAB-rainfed produced the mean yield of 2569.6 while the range was 1170.0 to 3439.0 (Table 4). Other statistical parameters like, standard deviation, standard error of mean, 1st, 2nd and 3rd quartiles for traits studied in growth chamber were also given in Table 4.

Mean values for all of the studied chickpea genotypes were given in Table 5. Genotypes 2 (CM776/06), 7 (CM848/06), 10 (09AK055), 23 (K0017–10), 24 (K0019–10), 25 (K0024–10), 26 (K0026–10), 29 (K0031–10), 30 (K0032–10), 31 (K0037–10), 32 (K0042–10), 37 (K0068–10), 38 (K0069–10), 39 (09AG019), 45 (08AG015), 54 (D0097–10) and 55 (D0099–10) showed the 100% GSI under growth chamber conditions. The lowest GSI was observed 33.33, 44.44 and 66.67% for genotypes 44 (09AG006), 50 (D0085–10) and 28 (K0030–10), respectively (Table 5). However, two chickpea genotypes i.e. 38 (K0069–10) and 39 (09AG019) showed the increased germination percentage having GSI value of 133.3%under lower osmotic stress conditions (Table 5).

**Table 5:** Physiological parameters^a^ and yield data of 55 genotypes of chickpea

		Seedling traits					Physio-chemical traits							Seed yield
Sr.No	Genotype	GSI (%)	RLSI (%)	SLSI (%)	FWSI (%)	DWSI (%)	CMS (%)	RWC (%)	ELWL (%)	ECCL (%)	Betain (μmol/g)	Proline (μmol/g)	NRA (μ mol NO_**2**_. g^**−1**^FW h^**−1**^)	Yield (kg ha^**−1**^)
1	CM709/06	77.77	20.72	35.43	16.92	26.72	88.87	97.65	8.00	6.07	1.65	2.69	2.41	1717
2	CM776/06	100.0	19.57	53.67	27.16	36.32	88.20	71.58	8.67	4.03	1.71	4.38	4.85	1745
3	CM1004/06	88.89	32.68	45.55	28.27	38.53	89.17	72.78	9.00	5.98	2.61	2.70	4.94	1808
4	CM687/06	88.89	40.16	95.90	31.97	37.87	83.63	71.05	8.00	6.94	1.74	23.23	4.87	1467
5	CM731/06	88.89	51.18	100.9	40.76	46.02	84.47	71.71	8.67	11.11	1.66	1.98	2.45	1562
6	CM795/06	88.89	41.55	134.1	35.59	41.33	88.17	120.0	9.00	9.92	1.37	2.16	3.49	1777
7	CM848/06	100.0	57.50	63.72	47.16	49.67	84.40	58.62	8.67	9.98	1.83	2.35	4.80	1543
8	CM1528/03	88.89	44.16	64.86	40.76	35.69	83.23	90.32	8.00	9.21	1.38	16.09	2.74	2043
9	2124	88.89	39.90	76.48	37.06	37.66	77.50	80.30	8.00	10.78	1.33	10.85	4.01	1853
10	09AK055	100.0	62.59	98.93	47.07	74.25	85.03	77.33	8.00	8.99	2.07	19.79	4.88	1993
11	2175	66.67	26.12	53.85	21.56	42.68	81.70	51.49	8.00	5.15	1.64	23.83	4.54	2073
12	2204	88.89	76.19	99.26	66.89	69.48	77.50	62.04	8.00	6.13	1.71	2.04	4.77	1326
13	K002–10	88.89	50.33	76.09	41.75	49.01	86.37	76.44	8.00	6.92	1.61	3.05	2.47	1802
14	K003–10	88.89	30.62	51.28	15.98	23.64	83.46	62.68	8.67	6.07	1.60	16.95	2.44	1633
15	K004–10	88.89	108.3	116.0	45.89	47.54	75.00	82.38	8.67	4.14	1.33	15.24	4.68	1323
16	K005–10	88.89	23.68	17.45	9.296	18.30	77.43	48.12	8.00	3.79	1.31	2.01	4.87	1937
17	K008–10	75.00	50.50	50.67	55.40	53.3	91.53	84.88	8.00	2.33	1.52	3.10	4.27	1965
18	K010–10	66.67	53.12	51.67	47.70	60.77	82.26	51.78	8.33	3.33	2.02	2.25	2.58	2081
19	K012–10	77.78	46.81	74.79	23.90	49.28	89.13	95.41	8.00	2.58	1.93	11.11	4.41	1550
20	K0013–10	88.89	32.01	54.31	20.17	28.61	85.40	50.80	8.00	2.77	1.37	12.24	2.65	1774
21	K0014–10	88.89	53.24	59.49	26.80	32.58	75.27	65.48	8.00	4.36	1.16	11.39	4.04	1652
22	K0016–10	66.67	14.43	42.25	13.65	19.44	84.23	115.8	8.00	3.95	1.81	13.13	4.82	1611
23	K0017–10	100.0	75.21	257.6	64.86	96.04	88.43	44.41	8.00	3.15	1.37	3.13	5.01	1514
24	K0019–10	100.0	60.23	106.9	76.67	104.2	93.73	64.84	9.00	2.21	1.81	7.26	4.88	1505
25	K0024–10	100.0	50.61	98.54	50.31	55.91	88.43	64.79	8.67	3.02	1.31	6.00	4.17	1376
26	K0026–10	100.0	93.76	47.94	33.19	42.86	87.90	86.81	8.00	2.91	2.32	8.21	4.29	1368
27	K0027–10	88.89	37.64	94.05	37.76	46.64	82.83	53.22	7.67	6.12	1.93	5.37	4.74	1628
28	K0030–10	66.67	25.85	35.33	20.12	25.34	82.95	71.05	8.67	2.22	1.15	7.93	4.96	1487
29	K0031–10	100.0	44.07	47.91	22.59	28.34	81.40	71.77	8.00	2.38	1.80	12.89	5.06	1754
30	K0032–10	100.0	79.26	102.4	68.00	58.33	88.63	70.65	8.00	4.13	2.15	23.51	4.94	1670
31	K0037–10	100.0	47.74	43.10	43.06	47.41	78.17	69.15	7.67	2.90	1.28	2.87	4.51	1217
32	K0042–10	100.0	32.58	23.55	24.25	35.86	80.70	71.23	8.67	2.95	1.86	2.43	4.86	1315
33	K0049–10	88.89	37.59	45.43	24.07	27.20	85.80	70.18	8.00	2.67	1.72	2.71	2.43	1430
34	K0052–10	77.78	47.68	44.52	30.18	33.82	88.43	69.37	7.67	3.11	1.31	2.26	4.60	1338
35	K0063–10	88.89	52.42	83.91	43.23	42.59	82.43	68.66	8.00	2.85	1.19	14.10	4.27	1285
36	K0066–10	88.89	42.64	47.54	41.81	43.52	86.60	71.97	8.33	2.87	1.82	18.91	4.58	1457
37	K0068–10	100.0	44.01	80.49	33.19	40.18	86.27	67.96	8.33	3.03	1.37	20.22	4.65	1347
38	K0069–10	133.3	39.00	81.56	32.34	46.23	82.62	69.41	8.00	2.83	1.69	2.37	4.70	1485
39	09AG019	133.3	98.21	110.6	59.61	59.90	95.47	62.86	8.33	9.59	1.71	3.25	4.35	1679
40	09AG015	88.89	45.11	33.19	34.44	36.35	80.67	75.00	8.00	3.56	1.83	2.21	4.21	1326
41	08AG004	88.89	43.39	37.54	19.30	28.28	91.20	66.67	8.00	4.65	1.31	2.35	5.01	1923
42	08AG016	66.67	27.27	86.67	20.86	25.32	82.23	66.02	8.00	5.91	1.32	5.11	4.33	1826
43	09AG002	88.89	39.15	114.6	30.67	38.84	93.40	72.22	8.00	4.73	1.21	2.16	4.54	1980
44	09AG006	33.33	8.421	20.8	4.356	5.94	86.37	73.86	8.00	6.57	1.41	2.03	4.47	1767
45	08AG015	100.0	54.26	106.2	24.51	34.00	87.67	66.10	8.00	20.91	1.87	2.71	2.65	1572
46	D0078–10	88.89	29.33	52.87	28.64	44.64	83.17	66.67	9.00	3.78	1.92	2.02	4.40	1803
47	D0079–10	55.55	21.78	48.61	10.27	15.31	83.23	60.33	8.00	3.45	1.75	1.79	2.25	1631
48	D0080–10	50.00	34.00	36.00	12.47	15.51	92.43	66.43	8.00	2.46	1.76	1.90	2.35	1968
49	D0081–10	55.55	49.40	77.08	8.038	16.43	83.67	64.54	8.00	2.26	1.53	10.49	4.87	1913
50	D0085–10	44.44	47.65	89.05	15.86	20.71	86.63	70.81	8.33	6.76	1.51	11.61	4.05	2015
51	D0091–10	50.00	113.0	90.70	25.00	39.26	91.18	68.84	8.00	2.17	1.29	2.08	4.70	2283
52	D0094–10	66.67	30.77	23.33	8.176	13.67	88.40	76.61	9.00	2.00	1.51	2.18	4.64	1575
53	D0096–10	66.67	39.21	43.6	25.66	37.29	83.67	68.71	8.00	3.43	2.23	17.73	4.21	1558
54	D0097–10	100.0	45.77	86.67	55.55	74.88	89.77	65.51	8.00	3.34	1.89	2.25	4.28	1911
55	D0099–10	100.0	62.91	36.32	18.739	25.29	83.27	64.67	8.00	3.23	1.32	3.26	4.64	1687

^a^*GSI* Germination stress tolerance index, *RLSI* Root length stress tolerance index, *SLSI* Shoot length stress tolerance index, *FWSI* Fresh weight stress tolerance index, *DWSI* Dry weight stress tolerance index, *CMS* Cell membrane stability, *RWC* Relative water content, *ELWL* Excised leaf water loss, *EC* Electrical conductivity

Genotypes K004–10, K0026–10, K0032–10, 09AG019 and D0091–10 had highest values for RLSI with means of 108.3, 93.76, 79.26, 98.21 and 113.0%respectively, whereas genotypes CM709/06, CM776/06, 2175, K005–10, K0016–10, K0030–10 and 09AG006 had the lowest RLSI 20.72, 19.57, 26.12, 23.68, 14.43, 25.85 and 8.42%, respectively (Table 5).GenotypesK0017–10, K0019–10, K0032–10, 09AG019, 09AG002 and 08AG015 have 257.7, 106.9, 102.4, 110.6and114.6%, respectively being the highest values for SLSI whereas, genotypes K005–10, K0042–10, 09AG006 and D0094–10 had the lowest values for SLSI 17.45, 23.55, 20.8, and 23.33%, respectively (Table 5). The lowest FWSI values were observed in 09AG006 (4.356%) followed by D0081–10 (8.038%),D0094–10 (8.176%), K005–10 (9.296%), D0079–10 (10.27%), K0016–10 (13.65%), K003–10 (15.98%), and CM709/06 (16.92%). The highest FWSI was observed in K0019–10 (76.67%), followed by K0032–10 (68.00%), 2204 (66.89%) and K0017–10 (64.86%) (Table 5). The maximum DWSI was observed for K0019–10 (104.2%), followed by K0017–10 (96.04%), D0097–10 (74.88%), 09AK055 (74.25%) and 2204 (69.48%) whereas, the minimum DWSI was observed in 09AG006 (5.94%), followed by D0094–10 (13.67%), D0079–10 (15.31%), D0080–10 (15.51%), D0081–10 (16.43%), K005–10 (18.30%) and K0016–10 (19.44%) (Table 5).

The highest value for CMS was observed in 09AG019 (95.47%), followed by K0019–10 (93.73%), 09AG002 (93.40%), D0080–10 (92.43%), K008–10 (91.53%), 08AG004 (91.20%) and D0091–10 (91.18%) whereas, the lowest CMS was observed in K0037–10 (78.17%), 2204 (77.50%), K005–10 (77.43%), K0014–10 (75.27%) and K004–10 (75.00%) (Table 5). The highest RWC was found in CM795/06 (120.0%), followed by K0016–10 (115.8%), CM709/06 (97.65%), K012–10 (95.41%), CM1528/03 (90.32%), K0026–10 (86.81%), whereas it was the lowest inK0017–10 (44.41%), followed by K005–10 (48.12%),K0013–10 (50.80%), and K0027–10 (53.22%), (Table 5). The highest ELWL value of 9.00% was observed in CM1004/06, followed by CM795/06, K0019–10, D0078–10 and D0094–10 whereas, the lowest 7.67% was observed in K0027–10,followed by K0037–10 and K0052–10 (Table 5).

The highest value of ECCL was observed in 08AG015 (20.91%), followed by CM731/06 (11.11%) and 2124 (10.78%) whereas, the lowest was in D0094–10 (2.00%) followed by D0091–10 (2.17%),K0019–10 (2.21%),K0030–10 (2.22%), D0081–10 (2.26%), K008–10 (2.33%), K0031–10 (2.38%), K012–10 (2.58%), K0013–10 (2.77%), and K0026–10 (2.91%), (Table 5).

The highest glycinebetaine (GB) contents were observed in CM1004/06 (2.61 μmol/g), followed by K0026–10 (2.32 μmol/g), D0096–10 (2.23 μmol/g), K0032–10 (2.15 μmol/g), 09AK055 (2.07 μmol/g), and K010–10 (2.02 μmol/g),, whereas, the lowest GB contents were found in K0030–10 (1.15 μmol/g), followed by K0014–10 (1.16 μmol/g), K0063–10 (1.19 μmol/g), K0024–10 (1.31 μmol/g), K005–10 (1.31 μmol/g), K004–10 (1.33 μmol/g) and 2124 (1.33 μmol/g) (Table 5).

Proline contents were the highest in 2175 (23.83 μmol/g), followed by K0032–10 (23.51 μmol/g), CM687/06 (23.33 μmol/g), and K0068–10 (20.22 μmol/g), whereas, it was the lower in D0079–10 (1.79 μmol/g) followed by D0080–10 (1.90 μmol/g), CM731/06 (1.98 μmol/g), K005–10 (2.01 μmol/g), D0078–10 (2.02 μmol/g), 09AG006 (2.03 μmol/g), 2204 (2.04 μmol/g), D0091–10 (2.08 μmol/g), CM795/06 (2.16 μmol/g),, and D0094–10 (2.18 μmol/g), respectively (Table 5).

The highest NRA was recorded in K0031–10 (5.06 μ mol NO_2_.g^− 1^FW h^− 1^), followed byK0017–10 (5.01 μ mol NO_2_.g^− 1^FW h^− 1^), and 08AG004 (5.01 μ mol NO_2_.g^− 1^FW h^− 1^) whereas the lower NRA was observed in D0079–10 (2.25 μ mol NO_2_.g^− 1^FW h^− 1^), followed by D0080–10 (2.35 μ mol NO_2_.g^− 1^FW h^− 1^), CM709/06 (2.41 μ mol NO_2_.g^− 1^FW h^− 1^) and K0049–10 (2.43 μ mol NO_2_.g^− 1^FW h^− 1^),respectively (Table 5).

Average grain yield across all of the studied environments showed that genotype D0091–10 (2283 kg ha^− 1^) maintained the highest yield followed by, K010–10 (2081 kg ha^− 1^), 2175 (2073 kg ha^− 1^), CM1528/03 (2043 kg ha^− 1^), and D0085–10 (2015 kg ha^− 1^) whereas, genotypeK0037–10 (1217 kg ha^− 1^) had the lowest grain yield followed byK0063–10 (1285 kg ha^− 1^) (Table 5).

### Principal component biplot analysis

**Fig. 1:**
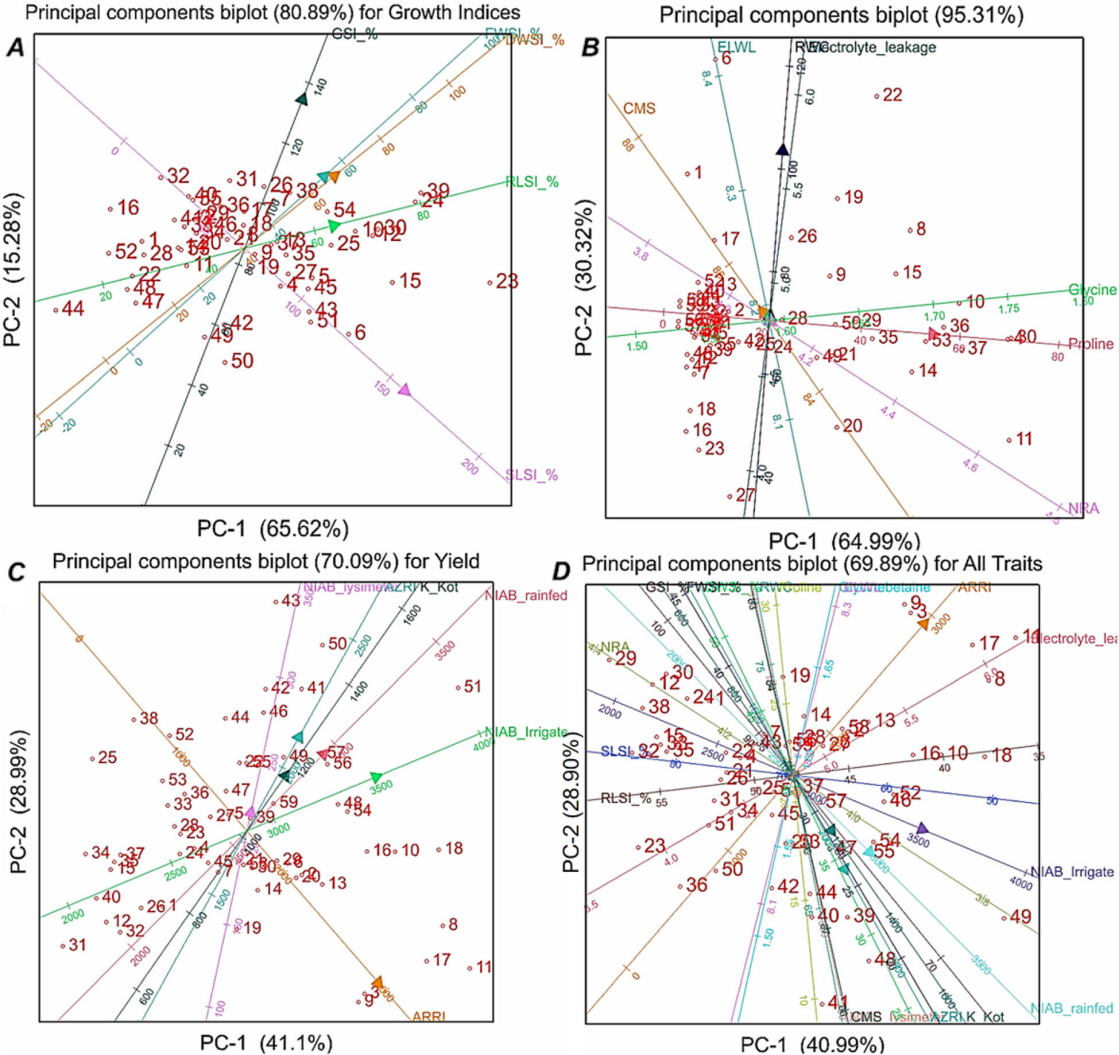
PCA biplot analysis for different chickpea genotypes. **a**: PCA biplot for chickpea genotypes at early growth stages, **b**: PCA biplot for chickpea genotypes for physiological and biochemical traits, **c**: PCA biplot for seed yield of chickpea genotypes at different environments, **d**: Combined PCA biplot for all of the studied traits Whereas, Where, RLSI: root length stress tolerance index, SLSI: shoot length stress tolerance index, FWSI: fresh weight stress tolerance index, DWSI: dry weight stress tolerance index, CMS: Cell membrane stability, ELWL: excised leaf water loss, NRA: Nitrite reductase activity, RWC: relative water contents, NIAB: Nuclear Institute for Agriculture and Biology, AARI: Ayub Agricultural Research Institute, K.Kot: Kaloor kot farm.

PCA biplots were generated for each experiment separately and cumulatively. PCA biplot for early growth indices depicted the 80.89% (PC1: 65.62%, PC2: 15.28%) of the variability in the raw data (Fig. 1). Angle of the trait vectors was reflecting the correlation of variables. If angle between two trait vectors is <90^o^ then correlation is positive, if angle is >90^o^ then correlation is negative while if equivalent to 90^o^ then traits are said be independent of each other. Growth indices were mostly had positive correlation except for the correlation of SLSI and GSI. FWSI and DWSI which have strong positive correlation. Traits vectors were labeled for the mean values of the corresponding traits which are facilitating the selection of genotypes for the lowest or the highest mean values. Among studied chickpea genotypes, 23 (K0017–10), 39 (09AG019), 24 (K0019–10), 15 (K004–10), 30 (K0032–10), 12 (2204), 10 (09AK055), 6 (CM795/06), 25 (K0024–10) and 54 (D0097–10) had the highest mean values and better performance while the genotypes 44 (09AG006), 47 (D0079–10), 48 (D0080–10), 52 (D0094–10), 16 (K005–10), 1 (CM709/06), 28 (K0030–10) and 22 (K0016–10) has the lowest mean values and relatively poor performaningfor studied indices of early growth stages (Fig. 1).

PCA biplot for physiological and biochemical traits of the chickpea genotypes was reflecting the 95.31% (PC1: 64.99%, PC2: 30.32%) of the total variability in data. Proline contents and GB contents had strong positive correlation between each other. Electrolyte leakage, RWC and ELWL were also positively correlated whereas, CMS and NRA were negatively correlated with each other. Among studied chickpea genotypes, 11 (2175), 4 (CM687/06), 30 (K0032–10), 37 (K0068–10), 36 (K0066–10), 10 (09AK055), 53 (D0096–10), 14 (K003–10), 8 (CM1528/03) and 15 (K004–10) had the highest NRA, GB and Proline contents. Genotypes 6 (CM795/06), 22 (K0016–10), 1 (CM709/06) and 19 (K012–10) had the highest values for electrolyte leakage, RWC, ELWL and CMS. Among all of the chickpea genotypes, 27 (K0027–10), 23 (K0017–10), 16 (K005–10), 18 (K010–10) and 7 (CM848/06) had the lowest mean values for all of the studied physiological and biochemical traits (Fig. 1).

PCA biplot for grain yield was reflecting the 70.90% of the total variability of yield data. AARI was proved to be entirely distinct environment, whereas, AZRI and K. Kot were positively correlated in their discrimination ability to assort the chickpea genotypes on yield performance. Genotypes 3 (CM1004/06), 9 (2124), 11 (2175), 17 (K008–10) and 8 (CM1528/03) were the highest yielding in AARI conditions. Genotypes 51 (D0091–10), 50 (D0085–10) and 18 (K010–10) were higher yielder across all of the environments. Genotypes 31 (K0037–10), 34 (K0052–10), 12 (2204), 32 (K0042–10), 26 (K0026–10), 1 (CM709/06), 40 (09AG015), 37 (K0068–10), 35 (K0063–10) and 15 (K004–10) were lower yielder across all of the environments except AARI (Fig. 1).

Cumulative PCA biplot for all of the studied traits showed the 69.89% (PC1: 40.99%, PC2: 28.90%) of the total variability in raw data. Wide spectrum distribution of trait vectors in this biplot showed that different traits of early growth stages, physio-chemical nature and yield performance have differential correlation with each other. GSI, FWSI, DWSI traits has positive correlation with RWC, Proline, GB, ELWL and yield at AARI environment. Yield performance at AARI environment was positively correlated with higher mean values of the some traits of early growth stages (GSI, FWSI and DWSI) and physiological and biochemical traits (RWC, Proline, GB, ELWL and Electrolyte leakage) background. Yield performance across all other environments, NIAB-lysimeter, NIAB-rainfed, AZRI, K. Kot had strong positive correlation with CMS. Yield performance of chickpea genotypes under NIAB-irrigated environment was distinctive from other environments due to higher vector angles. While, yield performance of subjected chickpea genotypes under AARI environment had negative correlation with reference to other environments (Fig. 1). Some of the traits atearly growth stages (GSI, FWSI, DWSI, SLSI, RLSI) and physio-chemical nature (Proline, RWC, NRA, ELWL, Glycine betaine and Electrolyte leakage) were negatively correlated with the yield performance of chickpea genotypes at AZRI, NIAB-rainfed, NIAB-NIAB-irrigated, NIAB-lysimeter and K. Kot environments (Fig. 1).

Among studied chickpea genotypes, 29 (K0031–10), 12 (2204), 30 (K0032–10), 38 (K0069–10), 24 (K0019–10), 1 (CM709/06), 32 (K0042–10), 15 (K004–10), 33 (K0049–10) and 35 (K0063–10) had higher mean performance for SLSI, RLSI, NRA, GSI, FWSI and DWSI however, these genotypes were lower in grain yield because these are allocated on the the lowest mean vector spoke of the yield environments. Chickpea genotypes 3 (CM1004/06), 9 (2124), 17 (K008–10), 11 (2175), 8 (CM1528/03) and 13 (K002–10) were high yielding under AARI environment whereas, genotypes 48 (D0080–10), 49 (D0081–10), 41 (08AG004), 39 (09AG019), 40 (09AG015) and 44(09AG006) were high yielding under NIAB-lysimeter, NIAB-rainfed, AZRI and K. Kot environments. Genotypes 49 (D0081–10), 18 (K010–10), 10 (09AK055) and 16(K005–10) were high yielding under NIAB-irrigated environment (Fig. 1).

### GGE biplot analysis

**Fig. 2:**
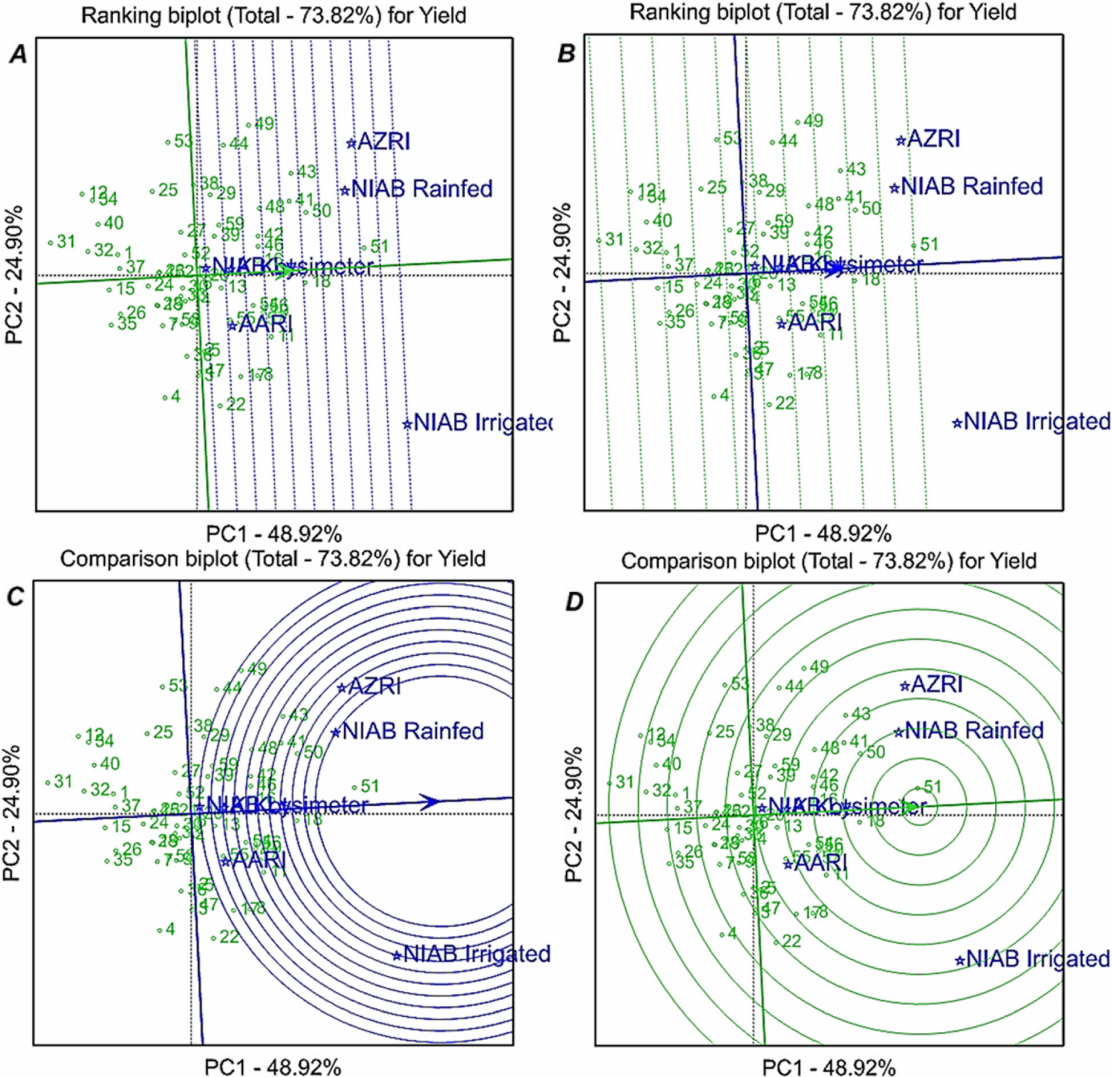
GGE biplot analysis for seed yield of different chickpea genotypes under different environments**. a**: Environment ranking GGE biplot for chickpea genotypes, **b**: Genotype ranking GGE biplot for seed yield of chickpea genotypes, **c**: Environment comparison GGE biplot for seed yield of chickpea genotypes, **d**: Genotype ranking GGE biplot for seed yield of chickpea genotypes. Where, RLSI: root length stress tolerance index, SLSI: shoot length stress tolerance index, FWSI: fresh weight stress tolerance index, DWSI: dry weight stress tolerance index, CMS: Cell membrane stability, ELWL: excised leaf water loss, NRA: Nitrite reductase activity, RWC: relative water contents, NIAB: Nuclear Institute for Agriculture and Biology, AARI: Ayub Agricultural Research Institute, K.Kot: Kaloor kot farm

Comparison biplots for environments and genotypes were generated by using the GGE biplot analysis. Environment and genotype comparison biplots were reflecting the 73.82% (PC1: 48.92% and PC2: 24.90%) of total variability in chickpea genotypes under subjected environments for grain yield. Arrow on average environment axis at the center of concentric circles showed the position of ideal environments to evaluate the performance of chickpea genotypes. Among studied environments, NIAB-rainfed environment was closer to the ideal environment followed by AZRI and NIAB-irrigated environments. NIAB-lysimeter and K. Kot were poor environments to evaluate the chickpea genotypes because these are positioned farthest away from the center of concentric circles (Fig. 2).

Arrow on the average environment axis at the center of concentric circles for genotypic comparison is reflecting the position of ideal genotype for subjected environments. Genotypes positioned closer to ideal genotype are preferable for selection under studied environments whereas, genotypes positioned farthest away from ideal genotype are poor performing based on the average yield performance across all environments. Genotype 51 (D0091–10), 18 (K010–10), 50 (D0085–10), 16 (K005–10), 46 (D0078–10), 42 (08AG016), 41 (08AG004), 48 (D0080–10), 43 (09AG002), 13 (K002–10) and 55 (D0099–10) were high yielding based on the average performance across all environments with reference to the ideal genotype. Genotypes 31 (K0037–10), 12 (2204), 34 (K0052–10), 40 (09AG015), 32 (K0042–10), 1 (CM709/06), 37 (K0068–10), 15 (K004–10), 26 (K0026–10) and 35 (K0063–10) were low yielding based on the average yield performance across all environments with reference to the ideal genotype (Fig. 2).

## Discussion

Drought stress is a global problem which adversely affects the performance of different crop plants. Development of drought tolerant cultivars is prerequisite to encounter the prevailing drought stress on sustainable basis. Evaluation of the chickpea responses to drought stress showed the significant variations for different traits of early growth stages, physio-chemical nature and grain yield. Significance of the variations depicted the differential responses of the genotypes to variable environments at different growth stages. These variations could be attributed to the differences in their genetic makeup of the studied genotypes. Different researchers have also reported the differential responses of the chickpea genotypes under different environments and at different growth stages ([1, 21, 22, 38] a, b [23];).

Different chickpea traits were targeted for identification of drought tolerant genotype among wide range of available germplasm. Literature indicated that evaluation and screening of germplasm under drought stress on the basis of physiological and biochemical parameters is prerequisite for crop improvement [38].GSI of different genotypes was unaffected, reduced or increased under water stress condition compared to the control treatment. Germination of the genotypes was affected differently under the provision of two different osmotic potentials (Table 5). Genotypes CM776/06, CM848/06, 09AK055, K0017–10, K0019–10, K0024–10, K0026–10, K0031–10, K0032–10, K0037–10, K0042–10, K0068–10, K0069–10, 09AG019, 08AG015, D0097–10 and D0099–10 showed the 100% GSI which showed their tolerance response under two different osmotic potentials for seed emergence. Genotypes 09AG006, D0085–10 and K0030–10 showed the most the lowest germination low osmotic potential whereas, genotype K0069–10 and 09AG019 showed the surprisingly increase in germination at lower osmotic potential. Such erratic behavior of germination percentage under stress condition (Table 5) also depends on seed viability, i.e., seed storing after harvesting, maintenance of seed moisture etc. [5, 6], so this type of seeds GSI could not show any linear or direct association with drought stress. The previous findings also suggested that only GSI could not be used for screening genotypes for drought tolerance [1].

Parameters of early growth stages were also used for evaluation of genotypic response under different water stress treatments. Growth parameters were studied as growth indices to reflect the relative value under both osmotic stress conditions provided by PEG solution. Different genotypes showed the differential (increased, decreased or unaltered) responses for these growth indices (FWSI, DWSI, RLSI and SLSI). Genotypes having the values for growth indices equal to 100, were showing the equivalent performance under both osmotic conditions whereas, values for growth indices near to zero are reflecting the highest reduction in performance under stress condition (Table 5). Genotypes having growth indices values more than 100are showing the better performance of genotypes for intended growth traits under stress condition (Table 5). Based on the buffering capacity to minimize the reduction of the different growth indices, genotypes 23 (K0017–10), 39 (09AG019), 24 (K0019–10), 15 (K004–10), 30 (K0032–10), 12 (2204), 10 (09AK055), 6 (CM795/06), 25 (K0024–10) and 54 (D0097–10) were osmotic stress tolerant at early growth stages. It was also previously reported that genotypic responses are variable under different stress conditions [26, 33] at early growth stages which indicate that consistent genotypic evaluation is prerequisite for development of new high yielding stress tolerant cultivars.

Tolerance at early growth stages is only useful if it harbored the high yield or it showed the positive association with other physio-biochemical traits. Therefore, performance of chickpea genotypes was further evaluated for physio-biochemical traits and yield performance. Significant variability in chickpea genotypes for different physiological and biochemical traits was evident in present study. Several other researchers have also reported the variability in physiological and biochemical traits of chickpea [2, 8, 11, 12, 16, 17]. Based on physiological and biochemical traits, genotypes CM687/06, K0032–10, K0068–10, K0066–10, 09AK055, D0096–10, K003–10, CM1528/03, K004–10, CM795/06, K0016–10, CM709/06 and K012–10 were better performer.

Genotypes with better performance at early growth stages were not necessarily having the better outcome for physio-chemical traits. Available findings indicated that, genotypes with better performance in physio-chemical traits not necessarily showed the better performance at early growth stages. Results of this study showed that among the better performing genotypes at early growth stages, 15 were higher in RWC and proline accumulation, 30 were higher in CMS, RWC and proline accumulation, 10 has higher glycine betain, proline, CMS and RWC whereas, 12 have lower proline and CMS values (Table 5). Researchers reported that higher proline, glycine betain, RWC and CMS values were reflecting the higher capability to tolerate the drought stress whereas, the lowest values for electrolyte leakage were depicting the capability for drought tolerance ( [2, 8, 11, 16, 17];).

Average yield across all of the subjected environments revealed that genotypes D0091–10, D0085–10, K010–10, 2175 and CM1528/03 were high in grain yield. Being high yielding under diverse and severe environmental conditions these genotypes can be declared drought tolerant. But these genotypes were not proved to be drought tolerant at early growth stages due to more reduction in growth performance at germination and seedling stages (Table 5). These genotypes were also not having extensively higher in physio-biochemical characteristics at pre-reproductive growth stages. These results showed that better performance of chickpea genotypes at early growth stages is not guaranteeing the high yield or drought tolerance at terminal growth stages. It may be corroborated that better growth performance at seedling stage under drought stress may induce the earliness in reproductive maturity which may confers yield penalty ([22] a,b [23, 27];).

PCA biplotis the most effective multivariate analysis evaluate the traits interaction and genotypic performance. PCA biplots analysis for SLSI, RLSI, NRA, GSI, FWSI and DWSI showed negative correlation with average grain yield of chickpea genotypes (Fig. 1). These findings further confirmed that selection of genotypes at early growth stages and based on different physio-chemical traits is not providing any surety for higher grain yield and terminal drought stress tolerance. PCA biplot was also extensively used by several researchers to dissect the traits correlation in different crop plants [7, 23–25].

Genotype × environment interaction (GEI) is seriously affected the performance of genotypes across different environments. Multi-environment trials are necessitating the manifestation of suitable biometrical tool to evaluate the interaction of genotypes with different environments, therefore, GGE biplot analysis was used in present study. In present study, NIAB-rainfed proved to be ideal environment for discrimination of chickpea genotypes for grain yield performance. Ideal environment is only referred on context based or theoretical perspectives but not on practical perspectives. NIAB-rainfed should be preferred for further studies to differentiate the genotypes for their yield performance. Among other environments, NIAB-lysimeter and K. Kot proved to be least capable of differentiating the genotypes for their yield performance (Fig. 2). Mean yield performance of chickpea genotypes was also very poor on NIAB-lysimeter and K. Kot environments due to very severe nature of water deficit at these environments. NIAB-lysimeter and K. Kot environments were depicting the similar genotypic performance therefore one of these environments could be excluded from further studies to cut short the management expenses and to alternatively add other environments in the studies. Maqbool et al .[22] also evaluated the chickpea genotypes under similar type of environments in different years and found the poor yield potential of lysimter conditions and better yield potential of NIAB-rainfed conditions.

GGE biplot analysis revealed that genotype D0091–10, K010–10, D0085–10, K005–10, D0078–10, 08AG016, 08AG004, D0080–10, 09AG002, K002–10 and D0099–10 were relatively superior in yield performance under drought stressed hotspot diverse environmental conditions (Fig. 2). Therefore, dissemination of these genotypes should be promoted in chickpea grown regions to have the higher yield potential under terminal drought conditions. Multi-environment hotspot evaluation of chickpea germplasm besides selection of high yielding genotypes also facilitates the identification of poor yielding or drought sensitive genotypes. Genotypes K0037–10, 2204, K0052–10, 09AG015, K0042–10, CM709/06, K0068–10, K004–10, K0026–10 and K0063–10 were poor yielding anddrought sensitive in present study according to multi-environment evaluation. These genotypes could efficiently be used as contrasting parents in different hybridization breeding programs intended for genetic improvement of chickpea genotypes for drought tolerance.

## Conclusions

Present investigation indicated that chickpea genotypes were significantly different for various seedling, physio-biochemical traits and yield performance across different environments. PCA biplot suggested that genotypes with better performance at early growth stages were not correspondingly high yielding. NIAB-rainfed environment proved ideal in nature to discriminate the chickpea genotypes whereas, NIAB-lysimeter and K. Kot was least discriminating the genotypes on yield basis. Based on the yield performance, genotypes D0091–10, K010–10, D0085–10, K005–10, D0078–10, 08AG016, 08AG004, D0080–10, 09AG002, K002–10 and D0099–10 were found to be high yielding and drought tolerant across multiple hotspot environments. These genotypes are intended for further evaluation for varietal approval to recommend for general cultivation on farmer fields in drought affected areas. Among physio-biochemical traits, higher proline, glycine betain, RWC and CMS were reflecting the higher capability to tolerate the drought stress in chickpea. Drought sensitive genotypes (K0037–10, 2204, K0052–10, 09AG015, K0042–10, CM709/06, K0068–10, K004–10, K0026–10 and K0063–10) were also identified in present study which are resourceful asset for using as contrasting parents in hybridization programs.

## Data Availability

The datasets used and/or analysed during the current study are available from the corresponding author on reasonable request.

## Abbreviations

NIAB: Nuclear Institute for Agriculture and Biology
NIFA: Nuclear Institute for Food and Agriculture
AZRI: Arid Zone Research Institute
AARI: Ayub Agriculture Research Institute
U.A.F: University of Agriculture
RWC: Relative water content
ELWL: Excised leaf water loss
PRI: Pulses Research Institute
PEG: Polyethylene glycol
MPA: Mega pascals
P.I.: Promptness index
RLSI: Root length stress tolerance index
SLSI: Shoot length stress tolerance index
FWSI: Fresh weight stress tolerance index
DWSI: Dry weight stress tolerance index
CMS: Cell membrane stability
EC: Electrical conductivity
GSI: Germination stress tolerance index
FW: Fresh weight
DW: Dry weight
R.H.: Relative humidity
NRA: Nitrite reductase activity
CRD: Completely randomized design
RCBD: Randomized complete block design
PCA: Principal Component Analysis
GEI: Genotype × environment interaction
GGE: Multi-environment trial
GB: Glycinebetaine
